# Virtual physiological analysis of non-culprit disease in patients with STEMI and multivessel disease: a substudy of the COMPLETE trial

**DOI:** 10.1093/ehjopen/oeaf057

**Published:** 2025-06-11

**Authors:** Gareth J Williams, Daniel J Taylor, Abdulaziz Al Baraikan, Hazel Haley, Mina Ghobrial, Matthew Knight, Kenneth Anigboro, Vignesh Rammohan, Rebecca Gosling, Tom Newman, Mark Mills, Rod Hose, David A Wood, John A Cairns, Chinthanie Ramasundarahettige, Rutaba Khatun, Helen Nguyen, Shamir R Mehta, Robert F Storey, Julian P Gunn, Paul D Morris

**Affiliations:** Division of Clinical Medicine, School of Medicine and Population Health, University of Sheffield, Beech Hill Road, Sheffield S10 2RX, UK; Division of Clinical Medicine, School of Medicine and Population Health, University of Sheffield, Beech Hill Road, Sheffield S10 2RX, UK; NIHR Sheffield Biomedical Research Centre, Sheffield Teaching Hospitals NHS Foundation Trust, Beech Hill Road, Sheffield S10 2RX, UK; Insigneo Institute for in Silico Medicine, Mappin Street, Sheffield S1 3JD, UK; Division of Clinical Medicine, School of Medicine and Population Health, University of Sheffield, Beech Hill Road, Sheffield S10 2RX, UK; College of Applied Medical Sciences, King Saud bin Abdulaziz University for Health Sciences, Prince Mutib Ibn Abdullah Ibn Abdulaziz Road, Riyadh, Saudi Arabia; Division of Clinical Medicine, School of Medicine and Population Health, University of Sheffield, Beech Hill Road, Sheffield S10 2RX, UK; South Yorkshire Cardiothoracic Centre, Sheffield Teaching Hospitals NHS Foundation Trust, Herries Road, Sheffield S5 7AU, UK; Division of Clinical Medicine, School of Medicine and Population Health, University of Sheffield, Beech Hill Road, Sheffield S10 2RX, UK; Division of Clinical Medicine, School of Medicine and Population Health, University of Sheffield, Beech Hill Road, Sheffield S10 2RX, UK; Faculty of Life Sciences and Medicine, King’s College London, Great Maze Pond, London SE1 1UL, UK; Division of Clinical Medicine, School of Medicine and Population Health, University of Sheffield, Beech Hill Road, Sheffield S10 2RX, UK; Division of Clinical Medicine, School of Medicine and Population Health, University of Sheffield, Beech Hill Road, Sheffield S10 2RX, UK; Division of Clinical Medicine, School of Medicine and Population Health, University of Sheffield, Beech Hill Road, Sheffield S10 2RX, UK; NIHR Sheffield Biomedical Research Centre, Sheffield Teaching Hospitals NHS Foundation Trust, Beech Hill Road, Sheffield S10 2RX, UK; Insigneo Institute for in Silico Medicine, Mappin Street, Sheffield S1 3JD, UK; South Yorkshire Cardiothoracic Centre, Sheffield Teaching Hospitals NHS Foundation Trust, Herries Road, Sheffield S5 7AU, UK; Division of Clinical Medicine, School of Medicine and Population Health, University of Sheffield, Beech Hill Road, Sheffield S10 2RX, UK; NIHR Sheffield Biomedical Research Centre, Sheffield Teaching Hospitals NHS Foundation Trust, Beech Hill Road, Sheffield S10 2RX, UK; Insigneo Institute for in Silico Medicine, Mappin Street, Sheffield S1 3JD, UK; South Yorkshire Cardiothoracic Centre, Sheffield Teaching Hospitals NHS Foundation Trust, Herries Road, Sheffield S5 7AU, UK; Division of Clinical Medicine, School of Medicine and Population Health, University of Sheffield, Beech Hill Road, Sheffield S10 2RX, UK; South Yorkshire Cardiothoracic Centre, Sheffield Teaching Hospitals NHS Foundation Trust, Herries Road, Sheffield S5 7AU, UK; Division of Clinical Medicine, School of Medicine and Population Health, University of Sheffield, Beech Hill Road, Sheffield S10 2RX, UK; Insigneo Institute for in Silico Medicine, Mappin Street, Sheffield S1 3JD, UK; Centre for Cardiovascular Innovation, University of British Columbia, Health Sciences Mall, Barton Street East, Vancouver, Canada BC V6T 1Z3; Centre for Cardiovascular Innovation, University of British Columbia, Health Sciences Mall, Barton Street East, Vancouver, Canada BC V6T 1Z3; Population Health Research Institute, Hamilton, Ontario, Canada L8L 2X2; Population Health Research Institute, Hamilton, Ontario, Canada L8L 2X2; Population Health Research Institute, Hamilton, Ontario, Canada L8L 2X2; Population Health Research Institute, Hamilton, Ontario, Canada L8L 2X2; Hamilton Health Sciences, McMaster University, Main Street West, Hamilton, Ontario, Canada L8S 4L8; Division of Clinical Medicine, School of Medicine and Population Health, University of Sheffield, Beech Hill Road, Sheffield S10 2RX, UK; NIHR Sheffield Biomedical Research Centre, Sheffield Teaching Hospitals NHS Foundation Trust, Beech Hill Road, Sheffield S10 2RX, UK; Insigneo Institute for in Silico Medicine, Mappin Street, Sheffield S1 3JD, UK; South Yorkshire Cardiothoracic Centre, Sheffield Teaching Hospitals NHS Foundation Trust, Herries Road, Sheffield S5 7AU, UK; Division of Clinical Medicine, School of Medicine and Population Health, University of Sheffield, Beech Hill Road, Sheffield S10 2RX, UK; NIHR Sheffield Biomedical Research Centre, Sheffield Teaching Hospitals NHS Foundation Trust, Beech Hill Road, Sheffield S10 2RX, UK; Insigneo Institute for in Silico Medicine, Mappin Street, Sheffield S1 3JD, UK; South Yorkshire Cardiothoracic Centre, Sheffield Teaching Hospitals NHS Foundation Trust, Herries Road, Sheffield S5 7AU, UK; Division of Clinical Medicine, School of Medicine and Population Health, University of Sheffield, Beech Hill Road, Sheffield S10 2RX, UK; NIHR Sheffield Biomedical Research Centre, Sheffield Teaching Hospitals NHS Foundation Trust, Beech Hill Road, Sheffield S10 2RX, UK; Insigneo Institute for in Silico Medicine, Mappin Street, Sheffield S1 3JD, UK; South Yorkshire Cardiothoracic Centre, Sheffield Teaching Hospitals NHS Foundation Trust, Herries Road, Sheffield S5 7AU, UK

**Keywords:** virtual FFR, FFR, CFD, Myocardial infarction, Percutaneous coronary intervention, Coronary angiography

## Abstract

**Aims:**

In the complete revascularization with multivessel PCI for myocardial infarction (COMPLETE) trial, staged complete revascularization in patients with ST-segment-elevation myocardial infarction (MI) reduced major adverse cardiovascular events compared with culprit-only revascularization. Inclusion was based on angiographic criteria.

**Objectives:**

We modelled non-culprit virtual fractional flow reserve (vFFR) and investigated interactions between physiological lesion severity and the benefits of complete revascularization in COMPLETE.

**Methods and results:**

All suitable angiograms from COMPLETE underwent software-based 3-dimensional (3D) arterial reconstruction and analysis of 3D-quantitative coronary angiography (QCA) and vFFR using computational fluid dynamics software. Physiological lesion significance was defined as vFFR ≤0.80 and was compared with operators’ visual angiographic analysis, 2D-QCA and 3D-QCA. vFFR was computed in 635 patients (710 lesions). 302 patients (48%) had ≥1 physiologically significant lesion and 333 (52%) had none. 321 (45%) lesions were physiologically significant and 389 (55%) were not. There was no statistically significant interaction between physiological lesion significance and any of the trial co-primary or key secondary clinical outcomes, or an exploratory outcome of ischaemia-driven revascularization without preceding MI (all interaction *P* > 0.30). 3D-QCA predicted vFFR significance more accurately than visual and 2D-QCA (concordance 73% vs. 49% vs. 59%, respectively).

**Conclusion:**

In this virtual physiological substudy of the COMPLETE trial, 52% of patients lacked any physiologically significant lesions and the benefits of complete revascularization appeared to be independent of physiological lesion significance. 3D-QCA was a better predictor of physiological significance than either 2D-QCA or operator visual analysis. Further research is warranted to compare angiography-guided and physiology-guided complete revascularization strategies.

## Introduction

For patients with ST-segment-elevation myocardial infarction (STEMI), evidence robustly supports immediate percutaneous coronary intervention (PCI) to open the occluded (culprit) artery.^1–3^ The decision whether to additionally intervene upon non-culprit stenoses has been extensively studied. Observational and randomized studies indicate that complete revascularization, with PCI to culprit and non-culprit stenoses, reduces adverse outcomes compared with a culprit-only approach.^4–10^

The complete vs. culprit-only revascularization strategies to treat multivessel disease after Early PCI for STEMI (COMPLETE) trial demonstrated that a complete revascularization approach led to a 26% relative reduction in cardiovascular (CV) death or new myocardial infarction (MI) and a 49% reduction in CV death, new MI, or ischaemia-driven revascularization (IDR), compared with a culprit-only strategy.^11,12^ In this trial, patients were eligible for randomization if the non-culprit lesion was ≥70% diameter stenosis by angiographic appearance. Angiographic appearance, however, is known to be a poor predictor of functional significance,^13–15^ and so it is likely that some patients with physiologically non-significant bystander disease were included and, conversely, some with functionally significant disease were excluded. The gold-standard invasive test for determining the functional significance of coronary stenoses is fractional flow reserve (FFR).^16^ FFR identifies ischaemia-causing disease with greater accuracy than angiography alone and is associated with improved clinical outcomes in patients with stable ischaemic heart disease.^17,18^ In the COMPLETE trial, FFR was indicated only for non-culprit lesions (NCLs) with 50–69% diameter stenosis, but this accounted for <1% of all cases. FFR and equivalent indices can now be computed from the angiogram, without a pressure wire. These methods calculate FFR using mathematical solutions, based on the laws of fluid dynamics, applied to 3-dimensional (3D)-reconstructed coronary anatomy, and have, therefore, become known as computed, angiographically derived or virtual FFR (vFFR). The first use of this technology was reported in 2013 and, since then, techniques have improved and been translated into clinical use, demonstrating similar efficacy to invasive FFR in guiding PCI during angiography.^19–21^ It is, therefore, possible to assess the functional significance of NCLs *post hoc*. The aims of this virtu-COMPLETE substudy were to analyse the physiological significance of NCLs included in the COMPLETE trial and determine whether vFFR had any association with the benefits of revascularization.

## Methods

### Study design and setting

The virtu-COMPLETE substudy was a *post hoc* analysis of the COMPLETE trial. The original COMPLETE trial protocol has been published in full.^11,12^ Briefly, patients were eligible for entry to the study if they demonstrated multivessel coronary artery disease during primary-PCI for STEMI, with at least one NCL that was deemed amenable to angioplasty. NCLs were identified as significant if judged to be ≥70% diameter stenosis by visual estimation during angiography, or if between 50–69% vessel diameter stenosis with an accompanying positive FFR measurement (≤0.80). Patients were randomly assigned to either a complete revascularization strategy, undergoing routine, staged PCI of all suitable NCLs regardless of clinical symptoms or evidence of ischaemia (*n* = 2016), or culprit-only management with no further revascularization after the primary-PCI procedure (*n* = 2025). Guideline-based medical therapy was recommended in both treatment groups. The first co-primary outcome was the composite of CV death or new MI, and the second co-primary outcome was the composite of CV death, new MI, or IDR. The key secondary outcome was the composite of CV death, new MI, IDR, unstable angina, or NYHA class IV heart failure. Median follow-up was 3 years. The trial received ethics committee approval from the Hamilton Integrated Research Ethics Board and ethics committee approval for each participating study centre. All patients gave informed consent.

### Study population, vFFR modelling and angiographic severity assessment

All the COMPLETE angiogram digital files (for both index and staged procedures) were transferred from the Population Health Research Institute, McMaster University, Hamilton, Canada to the Mathematical Modelling in Medicine Research Group, University of Sheffield, United Kingdom for analysis. All angiograms were first screened to ensure they contained the necessary digital imaging and communications in medicine (DICOM) data tags required for vessel reconstruction and vFFR assessment. Angiograms, which did not contain appropriate positioning, orientation, and image characteristic DICOM tags, were excluded. Suitable angiograms then underwent computational modelling of vFFR in non-culprit lesions, within proprietary software (VIRTUheart, University of Sheffield, UK).^19,20,22,23^ In brief, paired angiographic acquisitions, separated by ≥30°, were selected and used to reconstruct the arterial anatomy in 3D space, using an epipolar line-based, algebraic solution. The 3D reconstruction of coronary arterial anatomy relies upon there being at least two angiographic images, both showing the artery and lesion of interest, well opacified, with minimal foreshortening and overlapping vessels, during ECG-gated end-diastole.^24^ Angiograms that were of insufficient quality to model vFFR were excluded, and the reason for exclusion was documented. Cases were processed by one of eight experienced operators (G.J.W., D.J.T., A.A.B., H.H., M.G., M.K., K.A., and M.M.) who were blinded to the clinical outcomes, treatment allocation and, where applicable, the invasive FFR measurements. The 3D files representing the arterial luminal geometry then underwent computational fluid dynamics analysis to derive the trans-lesional pressure gradient, from which vFFR was calculated as *P*_d_/*P*_a_. For simulation boundary conditions, a personalized microvascular resistance was estimated from vessel dimensions, subtended myocardial mass and available demographic data.^19^ Invasively measured aortic pressure was used at the proximal boundary or was assumed to be 90 mmHg where unavailable.

For all successfully processed cases, the vFFR and the 3D-quantitative coronary angiographic (3D-QCA) stenosis severity were documented. The latter was calculated from the 3D reconstructed artery as: [reference vessel diameter—minimum (stenosis) vessel diameter]/reference vessel diameter. The reference vessel diameter was calculated as the average of the healthy inlet and outlet segment diameters. Operator-assessed angiographic severity and core-laboratory-assessed 2D-QCA were calculated previously.^25^ The former was assessed visually by the operator during the index procedure^11^ and the latter was analysed by core-laboratory from a single best projection with optimal opacification and minimal foreshortening and vessel overlap after calibration against the catheter tip diameter.^25^ Different methods of angiographic assessment yield different thresholds for significance. We applied the widely accepted thresholds for significance: for operator-assessed angiography this was ≥70% stenosis, as was used in the original COMPLETE trial,^11^ for 2D QCA was ≥60% stenosis, as was used in the QCA COMPLETE substudy,^25^ and for 3D-QCA was ≥50%, as was used in the FAVOR II trial of angiography-derived FFR.^26^

### Outcomes

Detailed definitions of the outcomes of the COMPLETE trial have been published.^11,12^ Deaths were categorized as CV or non-CV in nature. The study adjudication committee, who were blinded to treatment allocation, adjudicated all primary and secondary efficacy outcomes. To adjudicate an IDR outcome, cases were required to have CCS class 2 or more angina despite optimal medical therapy, intervention within 5 mm of the NCL that led to enrolment into the trial, and one or more of the following: reversible ischaemia on a non-invasive test for ischaemia; new ischaemic changes on electrocardiogram either at rest or on exertion; or an invasive FFR ≤0.80.

### Statistical analysis

Categorical variables are presented as frequency (percentage), normally distributed continuous variables as mean (± standard deviation) and non-normally distributed data as median [inter-quartile range]. Normality was assessed using the Kolmogorov–Smirnov test. Randomized COMPLETE patients with angiographic images suitable for accurate vFFR analysis were included in the analysis according to an intention-to-treat principle, using the co-primary and secondary outcomes data from the original study. Baseline patient, lesion and procedural characteristics were compared between groups using unpaired Student’s *t*-test and one-way ANOVA (F statistic) for parametric data, Wilcoxon rank-sum test (z statistic) and Kruskal–Wallis (H statistic) test for non-parametric data or the χ^2^ test for categorical data. Physiological significance was assigned to NCLs with vFFR ≤0.80. Patients with at least one physiologically significant NCL were assigned to one group, and those with NCLs that were all physiologically non-significant were assigned to another group. The effect of complete vs. culprit-only intervention on outcomes was estimated using a Cox proportional hazards models and interaction effects with a likelihood ratio test. Correlation between vFFR and angiographic lesion severity was assessed using Pearson’s correlation coefficient (continuous data) and Cohen’s kappa (dichotomized data). Cohen’s kappa adjusts for agreement expected by chance and is a number between −1.0 and 1.0 with values of 0, 0.10–0.20, 0.21–0.40, 0.41–0.60, 0.61–0.80, 0.81–0.90, and 1.0 indicating no (equivalent to chance), slight, fair, moderate, substantial, near-perfect, and perfect agreement, respectively.^27^ Negative values indicate agreement worse than that expected by chance. The association between angiographic severity and physiological significance was analysed by χ^2^ test. Statistical analyses were performed using SAS software (version 9.4, SAS Institute, Inc., Cary, North Carolina) and all figures were created using R (version 4.1.1). Statistical significance was accepted with an alpha level of ≤0.05, with all tests two-tailed. In view of the exploratory nature of the analyses, no correction was applied for multiple analyses.

## Results

### Case exclusions, patient, artery, and procedural characteristics

The majority of case exclusions were caused by inadequate angiogram DICOM tags (*n* = 2714) for vFFR analysis, accounting for 79.7% of all exclusions. A further 692 patients were excluded, primarily due to insufficient angiographic views for vessel reconstruction (*n* = 285, 8.4% of all exclusions, see supplementary for further details of exclusions). Therefore, 635 patients met the inclusion criteria (710 arteries). Of these, 323 patients (366 arteries) were in the complete revascularization group and 312 (344 arteries) were in the culprit-only group. The affected coronary artery was left anterior descending: 43.8%, right: 25.8%, left circumflex: 18.3%, obtuse marginal: 8.2%, diagonal or intermediate: 3.7% and left main: 0.3%. *Tables 1* and *2* detail the baseline patient, artery and procedural characteristics, according to vFFR severity and randomized allocation, and relative to the overall COMPLETE trial cohort. Baseline characteristics (demographics and comorbidities) between the COMPLETE main trial dataset and those included in the current substudy were comparable (see *Table 1*).

**Table 1:** Baseline characteristics of the main trial participants and the substudy cohorts

		vFFR Substudy							
	Original COMPLETE Trial	vFFR substudy −all	vFFR ≤ 0.80			vFFR > 0.80			
	All (*n* = 4041)	All (*n* = 635)	All (*n* = 302)	Culprit (*n* = 147)	Complete (*n* = 155)	All (*n* = 333)	Culprit (*n* = 165)	Complete (*n* = 168)	*P* Value
Age—year	62.0 (10.7)	62.3 (10.8)	62.4 (11.3)	61.7 (11.5)	63.1 (11.1)	62.2 (10.4)	62.2 (10.2)	62.1 (10.6)	0.75
Gender (male)—no. (%)	3225 (79.8)	507 (79.8)	233 (77.2)	105 (71.4)	128 (82.6)	274 (82.3)	135 (81.8)	139 (82.7)	0.11
Diabetes—no. (%)	787 (19.5)	117 (18.4)	65 (21.5)	30 (20.4)	35 (22.6)	52 (15.6)	26 (15.8)	26 (15.5)	0.06
Chronic renal insufficiency—no./total no. (%)	81 (2.1)	6 (1.0)	1/273 (0.4)	0/134 (0.0)	1/139 (0.7)	5/307 (1.6)	1/150 (0.7)	4/157 (2.5)	0.22
Prior myocardial infarction—no. (%)	302 (7.5)	50 (7.9)	22 (7.3)	12 (8.2)	10 (6.5)	28 (8.4)	12 (7.3)	16 (9.5)	0.60
Current smoker—no. (%)	1606 (39.7)	260 (40.9)	127 (42.1)	66 (44.9)	61 (39.4)	133 (39.9)	65 (39.4)	68 (40.5)	0.59
Hypertension—no. (%)	2009 (49.7)	297 (46.8)	138 (45.7)	69 (46.9)	69 (44.5)	159 (47.7)	74 (44.8)	85 (50.6)	0.60
Dyslipidaemia—no. (%)	1561 (38.6)	219 (34.5)	118 (39.1)	59 (40.1)	59 (38.1)	101 (30.3)	53 (32.1)	48 (28.6)	0.021
Prior PCI—no. (%)	283 (7.0)	44 (6.9)	20 (6.6)	11 (7.5)	9 (5.8)	24 (7.2)	9 (5.5)	15 (8.9)	0.77
Prior stroke—no. (%)	126 (3.1)	18 (2.8)	14 (4.6)	9 (6.1)	5 (3.2)	4 (1.2)	2 (1.2)	2 (1.2)	0.009
Body mass index (BMI)—kg/m^2^	28.3 (5.4)	28.0 (4.5)	28.2 (4.4)	27.9 (4.4)	28.4 (4.4)	27.9 (4.5)	27.8 (4.1)	28.0 (4.9)	0.50
Killip class ≥2—no./total no. (%)	430 (10.8)	56 (9.0)	28/293 (9.6)	15/140 (10.7)	13/153 (8.5)	28/327 (8.6)	19/161 (11.8)	9/166 (5.4)	0.67
Medications at discharge—no. (%)									
ASA	4026 (99.6)	629 (99.1)	298 (98.7)	143 (97.3)	155 (100)	331 (99.4)	164 (99.4)	167 (99.4)	0.43
P2Y_12_ inhibitor (any)	4021 (99.5)	631 (99.4)	300 (99.3)	146 (99.3)	154 (99.4)	331 (99.4)	165 (100)	166 (98.8)	>0.99
Ticagrelor	2579 (63.8)	449 (70.7)	222 (73.5)	107 (72.8)	115 (74.2)	227 (68.2)	120 (72.7)	107 (63.7)	0.14
Prasugrel	362 (9.0)	57 (9.0)	27 (8.9)	13 (8.8)	14 (9.0)	30 (9.0)	10 (6.1)	20 (11.9)	0.98
Clopidogrel	1088 (26.9)	126 (19.8)	51 (16.9)	26 (17.7)	25 (16.1)	75 (22.5)	35 (21.2)	40 (23.8)	0.08
Beta blocker	3580 (88.6)	572 (90.1)	270 (89.4)	129 (87.8)	141 (91.0)	302 (90.7)	150 (90.9)	152 (90.5)	0.59
ACEi/ARB	3437 (85.1)	566 (89.1)	271 (89.7)	128 (87.1)	143 (92.3)	295 (88.6)	144 (87.3)	151 (89.9)	0.64
Statin	3948 (97.7)	621 (97.8)	295 (97.7)	141 (95.9)	154 (99.4)	326 (97.9)	160 (97.0)	166 (98.8)	0.85
Haemoglobin A1c—%	5.8 (5.5–6.4)	5.9 (5.5–6.4)	5.9 (5.5–6.6)	5.9 (5.4–6.7)	5.9 (5.6–6.5)	5.8 (5.5–6.3)	5.8 (5.5–6.2)	5.8 (5.5–6.4)	0.13
LDL cholesterol—mmol/L	3.1 (1.2)	3.1 (1.4)	3.1 (1.3)	3.2 (1.6)	2.9 (0.9)	3.2 (1.5)	3.2 (1.3)	3.2 (1.7)	0.27
Peak creatinine—µmol/L	85.0 (28.9)	85.2 (28.6)	84.5 (30.4)	82.4 (19.5)	86.5 (38.0)	85.8 (26.8)	86.1 (23.1)	85.4 (30.1)	0.58

Baseline characteristics of the vFFR substudy are compared with those of the original COMPLETE trial (columns one and two). The other columns detail the baseline characteristics categorized by vFFR significance (≤0.80 vs. vFFR >0.80) and randomized group. *P* value is for any statistically significant between-group differences.

PCI, percutaneous coronary intervention; ASA, aspirin; ACEi, angiotensin converting enzyme inhibitor; ARB, angiotensin receptor blocker; LDL, low-density lipoprotein.

**Table 2:** Artery, lesion and procedural characteristics of the total COMPLETE trial population and the vFFR substudy cohorts

		vFFR Substudy							
	Original COMPLETE Trial	vFFR substudy all	vFFR ≤ 0.80			vFFR > 0.80			
	All (*n* = 4041)	All (*n* = 635)	All (*n* = 302)	Culprit (*n* = 147)	Complete (*n* = 155)	All (*n* = 333)	Culprit (*n* = 165)	Complete (*n* = 168)	*P* Value
Radial access—no. (%)	3263 (80.7)	531 (83.6)	244 (80.8)	127 (86.4)	117 (75.5)	287 (86.2)	140 (84.8)	147 (87.5)	0.07
Thrombus aspiration—no./total no. (%)	932 (24.9)	147 (26.2)	75/265 (28.3)	31/129 (24.0)	44/136 (32.4)	72/297 (24.2)	31/147 (21.1)	41/150 (27.3)	0.27
SYNTAX score									
STEMI culprit lesion specific score	8.7 (5.3)	8.4 (5.2)	7.9 (4.7)	7.7 (4.8)	8.1 (4.5)	8.8 (5.6)	9.0 (5.9)	8.6 (5.4)	0.020
Non-culprit lesion specific score	4.6 (2.7)	4.7 (2.5)	5.1 (2.6)	5.2 (2.6)	4.9 (2.7)	4.4 (2.4)	4.2 (2.3)	4.6 (2.5)	0.001
Baseline (including STEMI culprit)	16.2 (6.7)	16.1 (6.7)	16.4 (6.8)	16.2 (6.6)	16.6 (6.9)	15.9 (6.6)	15.7 (6.7)	16.0 (6.5)	0.33
Residual (after index PCI)	7.1 (4.8)	7.4 (4.7)	8.1 (5.0)	8.2 (4.8)	8.1 (5.1)	6.8 (4.3)	6.6 (4.4)	7.0 (4.1)	<0.001
Culprit lesion location—no./total no. (%)									
Left main	7 (0.2)	1 (0.2)	0/294 (0.0)	0/143 (0.0)	0/151 (0.0)	1/324 (0.3)	0/163 (0.0)	1/161 (0.6)	>0.99
Left anterior descending	1317 (34.1)	190 (30.7)	74/294 (25.2)	32/143 (22.4)	42/151 (27.8)	116/324 (35.8)	60/163 (36.8)	56/161 (34.8)	0.004
Circumflex	653 (16.9)	112 (18.1)	40/294 (13.6)	19/143 (13.3)	21/151 (13.9)	72/324 (22.2)	36/163 (22.1)	36/161 (22.4)	0.005
Right coronary artery	1881 (48.8)	315 (51.0)	180/294 (61.2)	92/143 (64.3)	88/151 (58.3)	135/324 (41.7)	67/163 (41.1)	68/161 (42.2)	<0.001
Number of residual diseased vessels—no./total no. (%)									
1	2950 (76.6)	455 (73.7)	203/293 (69.3)	102/142 (71.8)	101/151 (66.9)	252/324 (77.8)	122/163 (74.8)	130/161 (80.7)	0.017
≥2	901 (23.4)	162 (26.3)	90/293 (30.7)	40/142 (28.2)	50/151 (33.1)	72/324 (22.2)	41/163 (25.2)	31/161 (19.3)	0.017
Non-culprit lesion location (core lab)—no./total lesions (%)									
Left main	13 (0.2)	2 (0.2)	2/437 (0.5)	1/206 (0.5)	1/231 (0.4)	0/442 (0.0)	0/220 (0.0)	0/222 (0.0)	>0.99
Left anterior descending	2117 (39.5)	371 (42.2)	210/437 (48.1)	107/206 (51.9)	103/231 (44.6)	161/442 (36.4)	76/220 (34.5)	85/222 (38.3)	<0.001
Proximal LAD	541 (10.1)	97 (11.0)	47/437 (10.8)	24/206 (11.7)	23/231 (10.0)	50/442 (11.3)	28/220 (12.7)	22/222 (9.9)	0.79
Mid LAD	1213 (22.7)	232 (26.4)	136/437 (31.1)	72/206 (35.0)	64/231 (27.7)	96/442 (21.7)	40/220 (18.2)	56/222 (25.2)	0.002
Circumflex	1926 (36.0)	276 (31.4)	146/437 (33.4)	61/206 (29.6)	85/231 (36.8)	130/442 (29.4)	62/220 (28.2)	68/222 (30.6)	0.20
Proximal circumflex and obtuse marginal/ramus	1441 (26.9)	202 (23.0)	105/437 (24.0)	45/206 (21.8)	60/231 (26.0)	97/442 (21.9)	47/220 (21.4)	50/222 (22.5)	0.46
Distal left circumflex and posterior left ventricular branch	485 (9.1)	74 (8.4)	41/437 (9.4)	16/206 (7.8)	25/231 (10.8)	33/442 (7.5)	15/220 (6.8)	18/222 (8.1)	0.31
Right coronary artery	1299 (24.3)	230 (26.2)	79/437 (18.1)	37/206 (18.0)	42/231 (18.2)	151/442 (34.2)	82/220 (37.3)	69/222 (31.1)	<0.001

Artery, lesion, and procedural characteristics of the vFFR substudy are compared with those of the original COMPLETE trial (columns one and two). The other columns detail the baseline characteristics categorized by vFFR significance (≤0.80 vs. vFFR >0.80) and randomized group. *P* value is for any statistically significant between-group differences.

PCI, percutaneous coronary intervention; STEMI, ST-elevation myocardial infarction.

### Physiological results

The median vFFR was 0.82 [0.73–0.91], 0.82 [0.73–0.89], and 0.82 [0.73–0.91] for the total cohort, the complete revascularization group and the culprit-only group, respectively. On a per-patient analysis, of the 635 included patients, 302 patients (47.6%) had at least one physiologically significant lesion and 333 (52.4%) had no physiologically significant lesion. There was no difference in the proportion of patients with at least one physiologically significant lesion between culprit-only [147 (47.1%)] and complete revascularization [155 (47.9%)] groups (*P* = 0.83). On a per-lesion analysis, of the 710 lesions, 321 (45.2%) were physiologically significant and 389 (54.8%) were not. There was no difference in the proportion of physiologically significant lesions between the culprit-only [151 (43.9%)] and complete revascularization [170 (46.4%)] groups (*P* = 0.49).

### Association of vFFR with the benefits of complete revascularization

#### Co-primary outcomes

**Figure 1:**
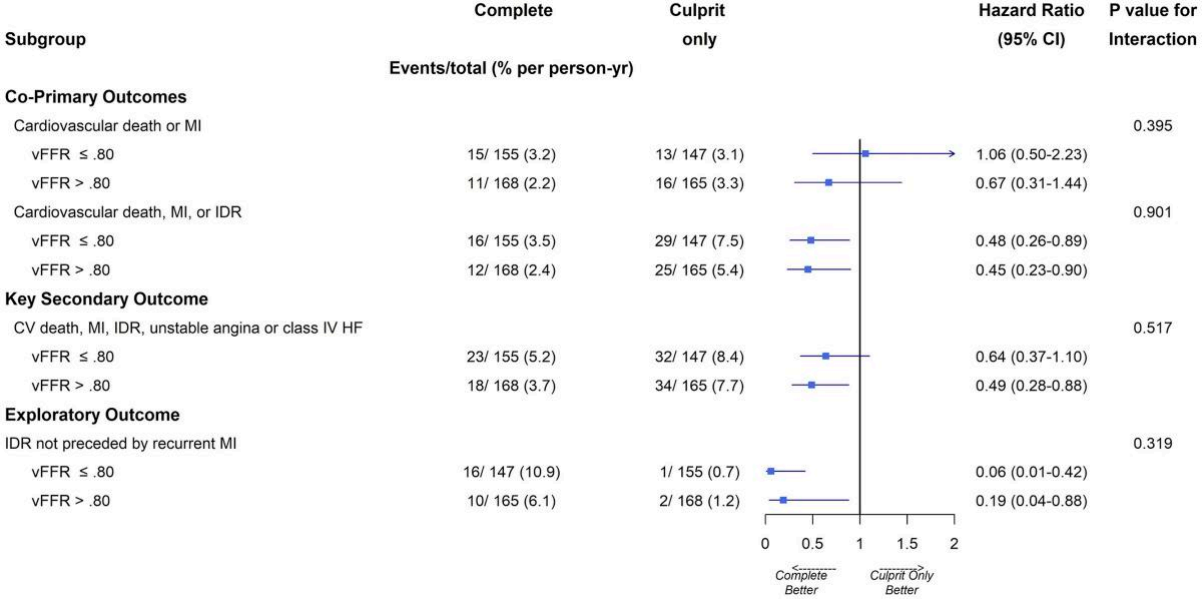
Forest plots showing the hazard ratios and 95% confidence intervals for effects of complete vs. culprit-only revascularization on the co-primary, key secondary and exploratory outcomes.

**Figure 2:**
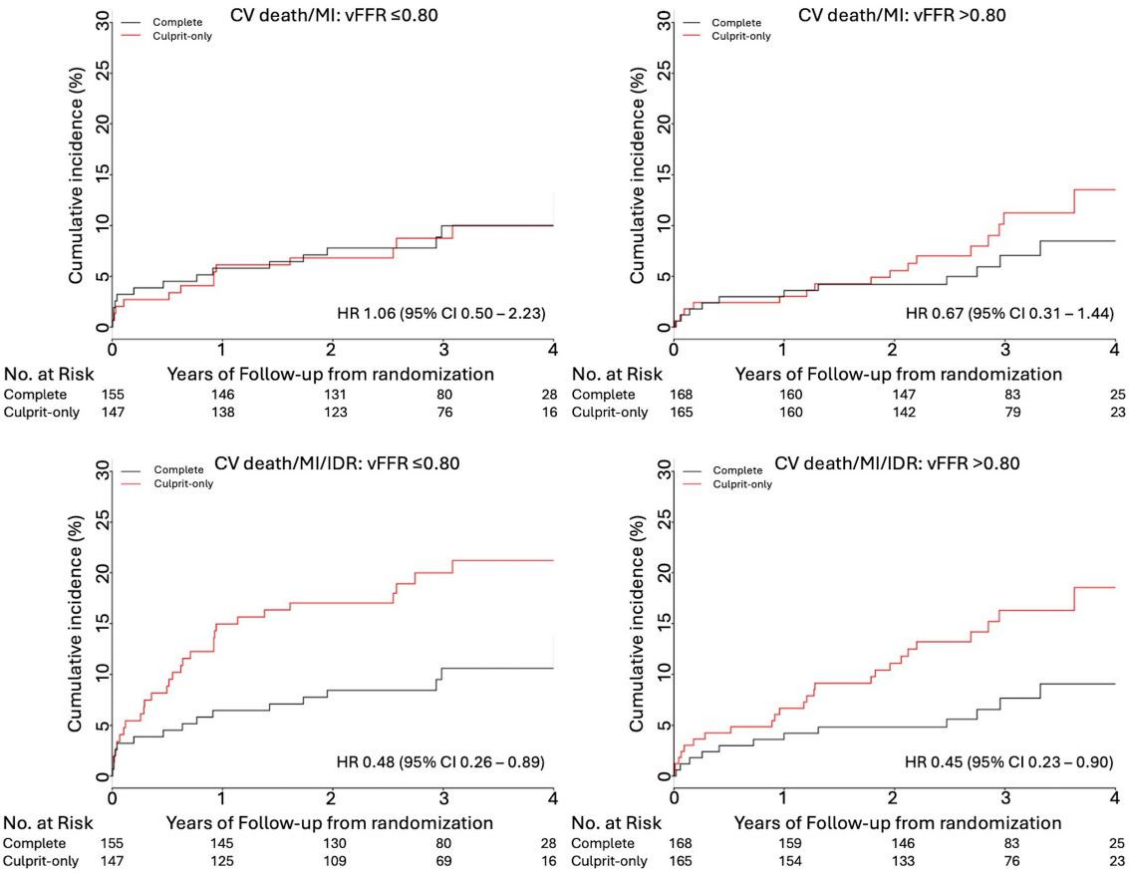
Cumulative incidence plots of the co-primary outcomes stratified by virtual physiology lesion significance. IDR, ischaemia-driven revascularization; MI, myocardial infarction.

There were no significant interactions for the effect of physiological lesion significance on either of the co-primary outcomes, or the key secondary outcome (*Figures 1* and *2*; *Table 3*). Among the 302 patients with vFFR ≤0.80, the incidence of the first co-primary outcome was 3.2% (per-person, per-year) in the complete group and 3.1% in the culprit-only group (HR 1.06; CI 0.50–2.23); in the 333 patients with no physiologically significant NCL, the incidence was 2.2% in the complete group and 3.3% in the culprit-only group (HR 0.67; CI 0.31–1.44), with no significant interaction for the effect of physiological NCL significance (*P* = 0.40). For the second co-primary endpoint of CV death, MI, or IDR, a strategy of complete revascularization was superior to a culprit-only strategy in both the physiologically significant group (3.5% vs. 7.5%, HR 0.48, CI 0.26–0.89) and in the physiologically non-significant group (2.4% vs. 5.4%, HR 0.45, CI 0.23–0.90), with no significant interaction for the effect of physiological NCL significance (*P* = 0.90).

**Table 3:** Clinical outcomes according to randomized group and vFFR substudy cohort

Clinical outcomes	Complete revascularization group	Culprit-only group	Hazard ratio (95% CI)	*P* value for interaction
Co-primary outcomes				
CV death or MI				0.395
vFFR ≤0.80	15 (3.2%)	13 (3.1%)	1.06 (0.50–2.23)	
vFFR >0.80	11 (2.2%)	16 (3.3%)	0.67 (0.31–1.44)	
CV death, MI, or IDR				0.901
vFFR ≤0.80	16 (3.5%)	29 (7.5%)	0.48 (0.26–0.89)	
vFFR >0.80	12 (2.4%)	25 (5.4%)	0.45 (0.23–0.90)	
Key secondary outcome				
CV death, MI, IDR, unstable angina, or class IV HF				0.517
vFFR ≤0.80	23 (5.2%)	32 (8.4%)	0.64 (0.37–1.10)	
vFFR >0.80	18 (3.7%)	34 (7.7%)	0.49 (0.28–0.88)	
Exploratory outcome				
IDR not preceded by recurrent MI				0.319
vFFR ≤0.80	16 (10.9%)	1 (0.7%)	0.06 (0.01–0.42)	
vFFR >0.80	10 (6.1%)	2 (1.2%)	0.19 (0.04–0.88)	

CV, cardiovascular; HF, heart failure; IDR, ischaemia-driven revascularization; MI, myocardial infarction; vFFR, virtual fractional flow reserve.

#### Key secondary outcomes

For the key secondary outcome (CV death, MI, IDR, UA or class IV heart failure), again, complete revascularization was superior to culprit-only in both the physiologically significant group (5.2% vs. 8.4%, HR 0.64, CI 0.37–1.10) and the physiologically non-significant group (3.7% vs. 7.7%, HR 0.49, CI 0.28–0.88), with no significant interaction for the effect of physiological NCL significance (*P* = 0.52).

#### Exploratory outcome

An exploratory analysis of the effect of physiological NCL significance upon the outcome of IDR without preceding MI was also performed. This was associated with a lower event rate when compared to a culprit-only strategy in both the physiologically significant group (0.21% vs. 4.03%, HR 0.06, CI 0.01–0.42) and the physiologically non-significant group (0.39% vs. 2.09%, HR 0.19, CI 0.04–0.88), with no significant interaction for the effect of physiological NCL significance (*P* = 0.32).

### Comparing operator-assessed, 2d-QCA and 3d-QCA angiographic severity with vFFR

Percentage NCL stenosis was significantly different when assessed visually, with 2D QCA and with 3D QCA (80 [70–90]% vs. 62.2 [54.4–70.7]% vs. 49.0 ± 12.4%; H = 1149, *P* < 0.0001). A similar relationship was true in the culprit-only (80 [70–80]% vs. 64.2 [54.4–71.3]% vs. 48.5 ± 12.3%, respectively; H = 728, *P* < 0.0001) and the complete revascularization groups (80 [70–85]% vs. 62.1 [54.4–70]% vs. 49.4 ± 12.5%, respectively; H = 807, *P* < 0.0001).

**Figure 3:**
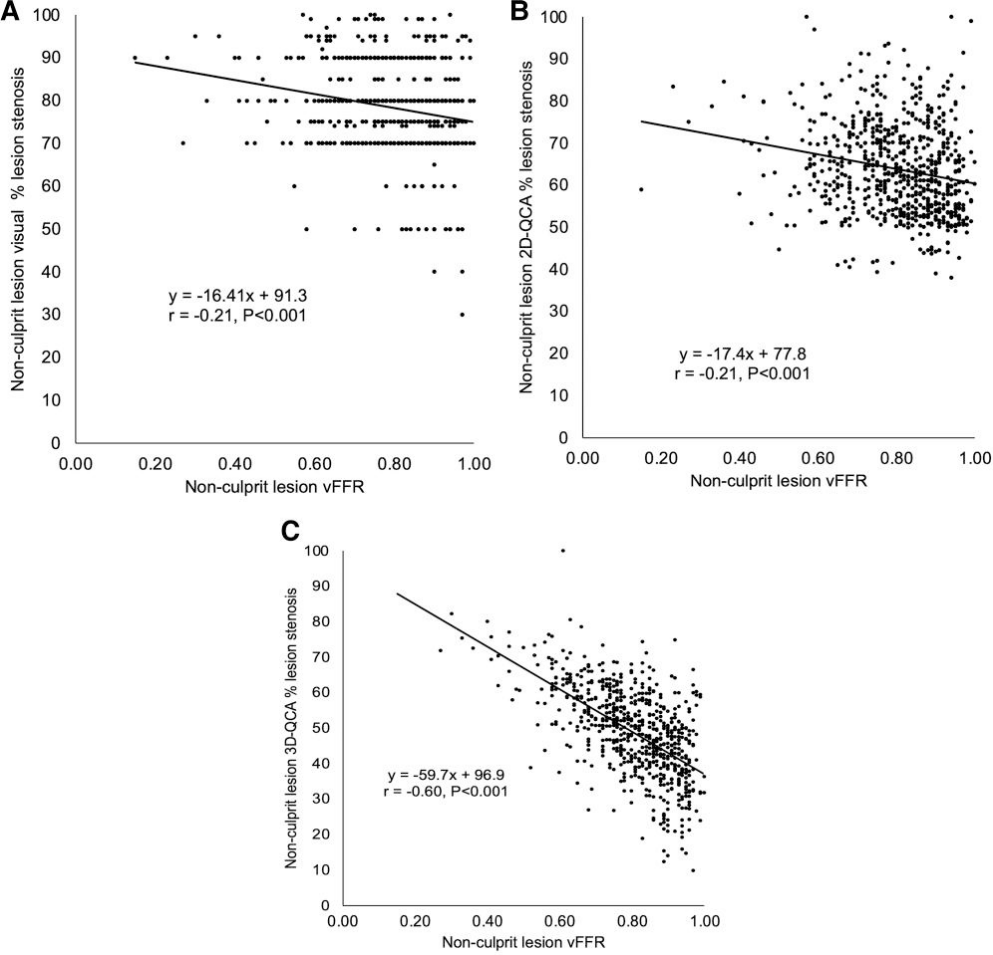
Scatter plots and lines of best fit for vFFR vs. operators’ visual angiographic severity (panel *A*), 2D-QCA (panel *B*), and 3D-QCA (panel *C*). QCA, quantitative coronary angiography; vFFR, virtual fractional flow reserve.

There was a weak but significant correlation between vFFR and operators’ visual angiographic severity (r = −0.21, *P* < 0.0001) (*Figure 3*, panel *A*). When these data were dichotomized into significant and non-significant, the Cohen’s kappa statistic was 0.054 (*P* < 0.001). In terms of predicting vFFR ≤0.80, the sensitivity, specificity, positive predictive value (PPV), negative predictive value (NPV), and overall accuracy of operator-assessed severity were 98%, 7%, 47%, 84%, and 48%, respectively. There was a weak but significant correlation between vFFR and 2D-QCA (*r* = −0.15, *P* < 0.0001) (*Figure 3*, panel *B*). When these data were dichotomized into significant and non-significant, the Cohen’s kappa statistic was 0.196 (*P* < 0.001). In terms of predicting vFFR ≤0.80, the sensitivity, specificity, PPV, NPV, and overall accuracy of 2D-QCA were 72%, 49%, 54%, 68%, and 59%, respectively. There was a stronger and significant correlation between vFFR and 3D-QCA (*r* = −0.60, *P* < 0.0001) (*Figure 3*, panel *C*). When these data were dichotomized into significant and non-significant, the Cohen’s kappa statistic was 0.465 (*P* < 0.001). In terms of predicting vFFR ≤0.80, the sensitivity, specificity, PPV, NPV, and overall accuracy of 3D-QCA were 74%, 73%, 69%, 78%, and 73%, respectively.

## Discussion

In this substudy of the COMPLETE trial, we analysed vFFR successfully in 635 patients (710 lesions). This is one of the largest randomized studies to date to investigate the influence of NCL physiology upon clinical outcome. There were four main findings. First, over half of all cases analysed (52%) had no physiologically significant NCL stenosis as assessed with vFFR. Second, there was no evidence of any significant interactions between vFFR and any of the co-primary, key secondary or exploratory outcomes. This is consistent with the theory that prognostic outcomes such as MI, IDR, and CV death are caused predominantly by atherosclerotic plaque rupture events, and that these events are more strongly related to plaque composition than to flow limitation. Third, compared with the operators’ visual assessment, lesion severity assessed by 2D- and 3D-QCA was significantly less severe, and fourth, physiological NCL significance assessed by vFFR correlated much better with 3D-QCA than either 2D-QCA or operators’ visual analysis. These findings reflect the subjectivity of angiographic analysis and the well-documented disconnect between angiographic appearance of coronary anatomy and physiology.^13–15^

Increasingly, the prognostic benefit of PCI is being demonstrated more in acute than chronic coronary syndromes.^28^ Physiological significance is a good predictor of the angina-causing potential of a coronary stenosis,^29^ but is just one of many features that may contribute to plaque instability and acute coronary syndromes. The risk of acute coronary syndromes is related to lesion instability, predicted by total atherosclerotic burden and features of plaque vulnerability such as an inflamed, thin-capped fibroatheroma, a lipid-rich or necrotic core, macrophage and lymphocyte infiltration, decreased smooth muscle cell content, and expansive remodelling.^30,31^ These features are better assessed with optical coherence tomography, intravascular ultrasound, and near-infrared spectroscopy.^32^ Minimum lumen area is also associated with plaque vulnerability and this, in turn, can be associated with flow restriction and reduced vFFR. Therefore, if a larger population had been studied, this association might have been observed in the analysis. However, it is important to remember that this is an association, and that flow is dependent not just on epicardial stenoses, but also on the distal microvascular bed. This is why physiological significance cannot be deduced purely from the epicardial artery anatomy. Indeed, if flow rate were dependent only on epicardial stenosis anatomy, the correlations between vFFR and QCA would likely be higher than those reported in the results.^33^

It is well established that operator assessment is subjective and frequently overestimates the true severity of coronary stenoses.^13–15^ Sheth *et al*. performed a 2D-QCA analysis of over 95% of the COMPLETE angiograms demonstrating that 35.6% of lesions in COMPLETE were <60% in diameter stenosis^25^ and that the benefits of complete revascularization were greater in those cases with stenosis severity of >60%. The current substudy demonstrated that lesion severity was downgraded serially from operator assessment to 2D-QCA, and from 2D-QCA to 3D-QCA (80.0% vs. 62.2% vs. 49.0%). Even QCA is not a good predictor of physiological significance.^14^ It is, therefore, interesting and clinically convenient that the benefits of complete revascularization, as demonstrated in the original COMPLETE trial, were based, first, on an anatomical criterion (as opposed to a physiological criterion) and, second, on the least objective angiographic assessment, namely an operator’s visual assessment of stenosis severity. This also suggests the possibility that the operators’ experienced eye may detect additional features that are hard to characterize or measure but may predict vulnerable lesions.

In the context of STEMI, the presence of multivessel disease is a common finding.^34^ A number of studies have suggested that complete revascularization may reduce major adverse cardiovascular events (MACE) when compared with a culprit-only strategy. However, observational studies can be affected by selection bias and confounding,^5,6^ and the benefits demonstrated in previous randomized trials have predominantly been driven by reduced rates of revascularization, as opposed to harder endpoints such as death and MI.^7–10^ Recent evidence from the PREVENT trial has however provided the strongest evidence yet to suggest PCI may reduce MACE secondary to vulnerable plaque rupture.^35^ In this trial, the benefit of PCI was proposed to be conferred by a functional thickening of the fibrous cap secondary to neointimal proliferation around stent struts. It is possible this was also true for the COMPLETE cohort, who may have also suffered with unstable plaque in non-culprit arteries and may explain why virtual physiological significance was not associated with clinical outcomes. Although meta-analyses had suggested a possible advantage with complete revascularization in terms of mortality or MI,^36–38^ the COMPLETE trial was the first randomized controlled trial adequately powered to detect the reduction in death or MI. In COMPLETE, > 99% of patients were recruited on the basis of their angiographic findings rather than physiological NCL significance. Physiological NCL significance was used in the Complete revascularization vs. treatment of the culprit lesion only in patients with ST-segment-elevation MI and multivessel disease (DANAMI-3—PRIMULTI) study and the fractional flow reserve-guided multivessel angioplasty in myocardial infarction (COMPARE-ACUTE) study.^9,10^ In both studies, FFR was used to guide revascularization, and both studies demonstrated reduced major adverse events in the complete revascularization group.

Given that COMPLETE did not use physiological NCL significance as the arbiter of inclusion, two questions arise. First, what is the optimal method to determine which NCLs to revascularize (angiographic or physiologically guided)? Second, would the superiority of complete revascularization, as demonstrated in the COMPLETE trial, have been strengthened or weakened had FFR been used as the arbiter of inclusion? The first question was tested in the Multivessel PCI Guided by FFR or Angiography for Myocardial Infarction (FLOWER-MI) trial, which reported after the COMPLETE trial. In this trial, patients with STEMI and multivessel disease received complete revascularization guided either by FFR or angiography.^39^ At 12 months follow-up (compared with 3 years in COMPLETE), there was no significant difference in the primary outcome (composite of any cause death, MI or urgent revascularization) between the groups. The authors also reported that the wide confidence intervals for the estimate of effect precluded a conclusive interpretation. Whilst the FFR-guided strategy was not shown to be superior, it was associated with a significantly reduced rate of stent insertion per-patient (1.01 vs. 1.50) for the same level of risk, suggesting that FFR may be useful in rationalizing intervention in this context.

The second question was addressed in a subsequent network analysis of eleven randomized trials and 8195 patients, in which complete revascularization was associated with a lower incidence of adverse events than a conservative approach, with no difference between an angiographic- or FFR-guided strategy.^40^ Overall, physiological NCL significance did not influence the benefits of complete revascularization when compared with a culprit lesion only strategy. Our exploratory analysis, examining the influence of NCL significance upon the incidence of ischaemia-driven revascularization, not preceded by MI, was equally negative. Our substudy was underpowered to detect a difference in this component of the second co-primary outcome. This will be investigated with greater statistical power in the COMPLETE-2 trial (NCT05701358), in which 5100 patients with STEMI and multivessel disease will be randomized to either physiologically guided or angiographically guided revascularization of NCLs.

Although this is one of the largest studies of the effects of physiology on the benefits of complete revascularization, a limitation was the high number of exclusions relative to the original study. These were driven by the technical requirements of computing vFFR. Nevertheless, 635 randomized patients and 710 lesions were analysed, and the two groups were well balanced. Case exclusions were mainly due to a lack of DICOM data fields which are required for 3D vessel reconstruction. Export of these data depends on local angiographic settings and not upon any case- or patient-specific factors, so there should not be significant bias arising from this. Even in cases with appropriate DICOM data, identifying images that are optimal for modelling can be challenging, hence the further attrition in the final analysis. Given that the images in COMPLETE were being acquired in the context of an acute STEMI, in which the priority is to open the culprit artery expeditiously, it is understandable that some of the studies of the non-culprit lesions were limited. Notwithstanding these exclusions, the substudy is comparable in size to other major studies that have investigated the influence of physiology on NCLs, including the PRAMI (*n* = 465),^7^ CvLPRIT (*n* = 296),^8^ and DANAMI-3-PRIMULTI (*n* = 627)^9^ trials. In addition, FRAME-AMI recruited 563 patients (less than half of the recruitment target of *n* = 1292).^41^ Despite the modest cohort size of FRAME-AMI, FFR-guided PCI was superior to angiography-guided PCI in the context of non-culprit disease.

In a previous substudy of the COMPLETE trial,^25^ there was an interaction between severity of stenosis on 2D-QCA and the benefits of complete revascularization. In the current substudy, there was an association between stenosis severity and physiological significance, which was strongest with 3D-QCA. Consequently, the lack of statistically significant interaction between vFFR and the benefits of complete revascularization might be due to inadequate power in our substudy to detect a weak interaction. A further limitation of the present substudy is that vFFR is a surrogate of invasive FFR and our results may not be representative of other angiography-derived FFR tools. Although vFFR provides a useful approximation of FFR, it cannot be 100% equivalent^33^ as has been recently demonstrated in a large trial of angiography-derived vs. invasive FFR.^42^ Invasive FFR measurement rates were very low in the original COMPLETE trial (<1%) and so there were insufficient cases to compare vFFR with FFR in the current substudy. The results of this substudy are, therefore, hypothesis-generating. Invasive FFR is being utilized in the COMPLETE-2 trial, which is recruiting both STEMI and NSTEMI patients.

## Conclusions

In this substudy of the COMPLETE trial, 52% of cases lacked physiological significance, as determined by vFFR. The clinical benefits of complete, vs. culprit-only revascularization, were independent of vFFR-determined physiological significance. Modelled physiological significance correlated better with 3D-QCA, than with either 2D-QCA or operators’ visual analysis. Further research is required to address the role of FFR in guiding revascularization of NCLs.

## Supplementary Material

oeaf057_Supplementary_Data

## Lead author biography

Gareth J. Williams is a cardiology registrar at the Aneurin Bevan Health Board, Wales. He completed his post-graduate research training during his scholarship with the University of Sheffield. His academic interests are in computational modelling and wearable technology, and their application to the assessment of patients with CV disease.
