# Supplementary material for: Virtual physiological analysis of non-culprit disease in patients with STEMI and multivessel disease: a substudy of the COMPLETE trial
Source: Eur Heart J Open. 2025 Jun 11;5(3):oeaf057. doi: 10.1093/ehjopen/oeaf057 (PMC12152305; doi:10.1093/ehjopen/oeaf057)
Supplement: oeaf057_Supplementary_Data [file oeaf057_supplementary_data.docx]

**Supplementary Material**


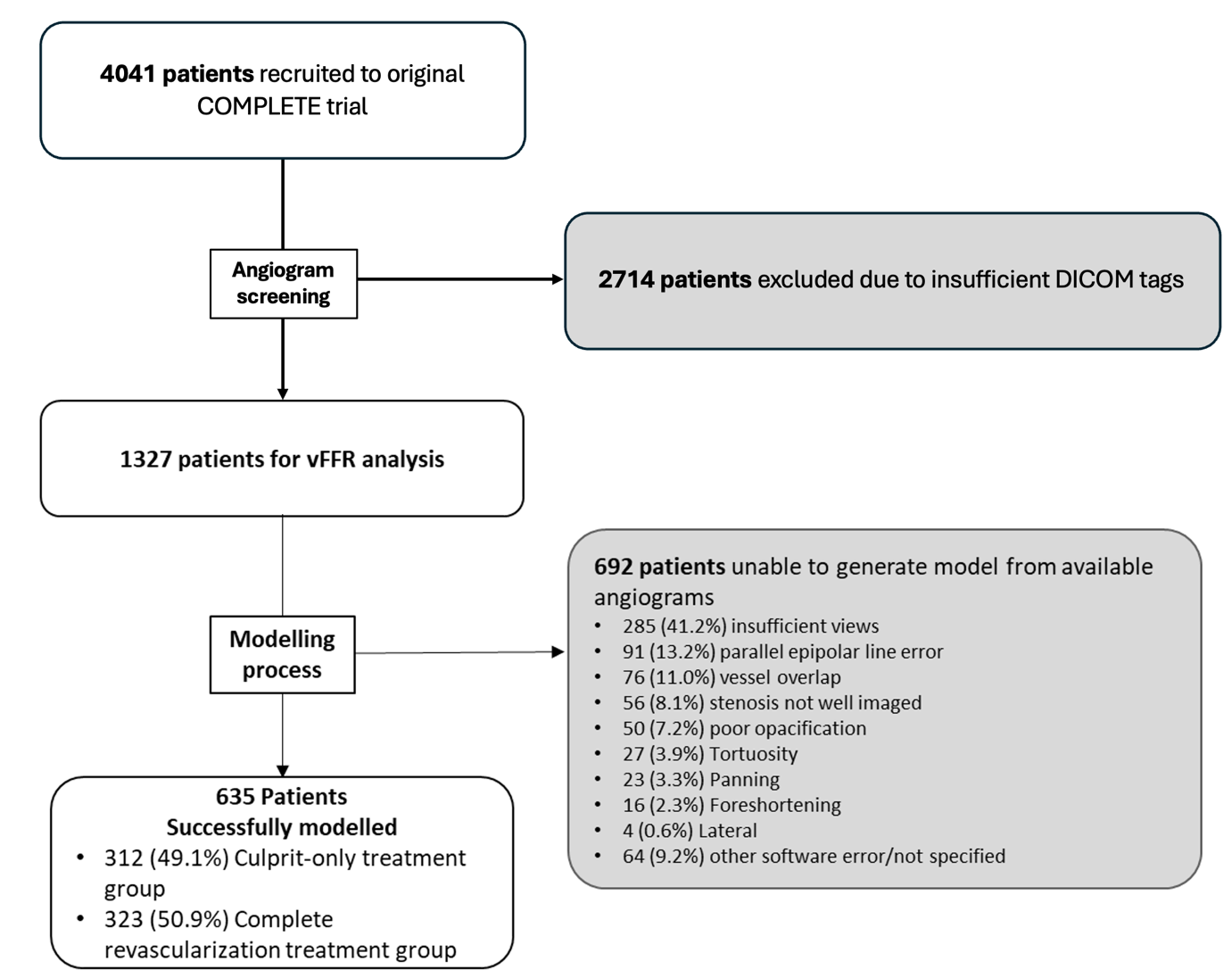


As detailed in figure (S1), of the 1327 patients with appropriate DICOM data, 692 had angiograms of insufficient quality for accurate vFFR analysis. The main reasons for exclusion were insufficient angiographic projection angles (41%) and inappropriate epipolar line angulation (13%). The final analysis included 635 patients and 710 individual lesions.
